# Supplementary figures and images for: Output voltage control of DAB converters based on uncertainty and disturbance estimation
Source: PeerJ Comput Sci. 2024 Jul 1;10:e2175. doi: 10.7717/peerj-cs.2175 (PMC11232625; doi:10.7717/peerj-cs.2175)

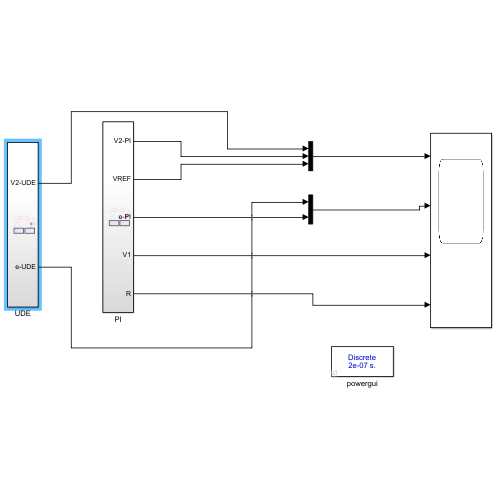

Supplement: Supplemental Information 2 — The file is opened using MATLAB R2022a. After opening the slx file, click the Run button in the menu bar and it. Open the oscilloscope to display the model health status, which shows the expected voltage Vref step transformation, input voltage step change, load resistance step change, load resistance step change, load resistance continuous periodic change of the system changes. After the oscilloscope image is saved, it is the image displayed in the TIF file, and after processing, it is the image in the article. [file peerj-cs-10-2175-s002.slx › metadata/thumbnail.png]

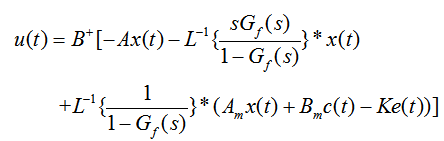

Supplement: Supplemental Information 2 — The file is opened using MATLAB R2022a. After opening the slx file, click the Run button in the menu bar and it. Open the oscilloscope to display the model health status, which shows the expected voltage Vref step transformation, input voltage step change, load resistance step change, load resistance step change, load resistance continuous periodic change of the system changes. After the oscilloscope image is saved, it is the image displayed in the TIF file, and after processing, it is the image in the article. [file peerj-cs-10-2175-s002.slx › simulink/resources/mwimg_24db90e1-de55-40e6-8541-1d736b2b55d0.png]
